# A new transcript in the *TCRB* locus unveils the human ortholog of the mouse pre‐*Dß1* promoter

**DOI:** 10.1002/iid3.172

**Published:** 2017-05-15

**Authors:** Bernard Lethé, Sylvia Snauwaert, Orian Bricard, David Schröder, Tiphanie Gomard, Gérald Hames, Catherine Muller, Christophe Lurquin, Emilie Gauthy, Ahmed Essaghir, Bart Vandekerckhove, Pierre G. Coulie

**Affiliations:** ^1^ Ludwig Institute for Cancer Research Brussels Belgium; ^2^ de Duve Institute Université catholique de Louvain Brussels Belgium; ^3^ Department of Clinical Chemistry, Microbiology and Immunology Ghent University Ghent Belgium

**Keywords:** Allelic inclusion, human *TCRB* locus, locus accessibility, PDß1 germline transcripts, T cells, thymocytes, *TCRB* transcripts

## Abstract

**Introduction:**

While most transcripts arising from the human T Cell Receptor locus reflect fully rearranged genes, several germline transcripts have been identified. We describe a new germline transcript arising from the human *TCRB* locus.

**Methods:**

cDNA sequencing, promoter, and gene expression analyses were used to characterize the new transcript.

**Results:**

The new germline transcript encoded by the human *TCRB* locus consists of a new exon of 103 bp, which we named *TRBX1* (*X1*), spliced with the first exon of gene segments *Cß1* or *Cß2*. *X1* is located upstream of gene segment *Dß1* and is therefore deleted from a V‐DJ rearranged *TCRB* locus. The *X1‐Cß* transcripts do not appear to code for a protein. We define their transcription start and minimal promoter. These transcripts are found in populations of mature T lymphocytes from blood or tissues and in T cell clones with a monoallelic *TCRB* rearrangement. In immature thymocytes, they are already detectable in CD1a^−^CD34^+^CD4^−^CD8^−^ cells, therefore before completion of the *TCRB* rearrangements.

**Conclusions:**

The *X1* promoter appears to be the ortholog of the mouse pre‐Dß1 promoter (*PDß1)*. Like *PDß1*, its activation is regulated by *Eß* in T cells and might facilitate the *TCRB* rearrangement process by contributing to the accessibility of the *Dß1* locus.

## Introduction

T cell receptor (TCR) gene rearrangements are complex multistep processes occurring at different stages of thymocyte maturation. They involve double‐strand DNA breaks at the recombination signal sequences that border the *V(D)J* gene segments and that are recognized by the RAG1/RAG2 recombinases 1. The process is tightly controlled, with the *TCRB* locus being rearranged before *TCRA*. For *TCRB* it occurs in two steps. First, at the CD4^−^CD8^−^ double negative stage a *Dß* gene segment recombines with a *Jß* segment yielding a *Dß.Jß* partially rearranged genomic DNA. These early rearrangements occur at the CD1^+^CD34^+^ stage. In a second step, ending before the CD4^+^CD8^+^ double positive (DP) stage, a *Vß* gene segment recombines with *Dß.Jß* 2. If this rearrangement leads to the production of a complete TCRB chain, the latter dimerizes with the pre‐TCRA chain and initiates ß‐selection. During ß‐selection, the pre‐TCR signaling suppresses RAG1/2 expression, induces several rounds of division and differentiation toward CD4^+^CD8^+^ DP thymocytes. At the DP stage, reexpression of RAG1/2 enables *TCRA* rearrangements to produce TCR‐positive DP cells 3.

Our previous work on T lymphocytes infiltrating human melanoma tumors led us to construct *TCRB* cDNA libraries 4. Starting from tumoral RNA, we first used a SMART‐PCR on cDNA extended from a Cß primer, and cloned the amplified products. Sequencing these products provided information on frequencies of tumor‐specific cytolytic T cell clones present in the tumor 4. A significant proportion of the sequences corresponded to a new *TCRB* germline transcript that we describe here.

## Materials and Methods

### Construction of *TCRB*‐targeted cDNA libraries

These libraries 4 were built up from RNA reverse‐transcribed with an antisense *Cß* primer [nt 63–47 of exon 1] in the presence of SMART II (Clontech®, Mountain View, CA, USA) 5, an oligonucleotide engineered to be copied at the 3′‐end of the growing cDNA during reversion, ought to an intrinsic Terminal deoxynucleotidyl Transferase (TdT) activity of the RT. A RNaseH^−^ RT‐enzyme in an appropriate buffer is needed for this 3′‐extension of the cDNA. With a primer consisting in the core of the SMART primer (5′‐gcagtggtaacaacgcagagta) and a *Cß* primer (primer 2 of Table 1) located near the 5′ end of *Cß*, the cDNA was amplified by PCR for a limited number of cycles with an annealing step at 60°C. Products shorter than 150 bp were removed by a Sepharose CL‐6B size‐exclusion column (Pharmacia‐Amersham). Under these conditions, the products derived from full length *TCRB* (*TRB‐LVDJC*) transcripts are in the range of 480 bp with 33 nucleotides coming from the primed region of *Cß*. Using a small fraction of the extracted RNA, in order to obtain the “smarted” cDNA from 0.5–1% of the *TCRB* transcripts, allows to estimate the frequency of the most prevalent clonotypes present in that sample, assuming a copy number of 200 productive *TRB‐LVDJC* transcripts/T cell. The method also allows to readily define the 5′‐ends of the *TCRB* transcripts, as an alternate 5′‐RACE approach, since many of the isolated clones represent full length cDNA.

**Table 1:** Primers used for *X1‐Cß* transcripts quantification

Sequence	Target	Position
5′‐GCTCAAAACATCCTGAGGACA (#1 in Fig. 2A)	*X1* sense	73–93^a^
5′‐CGACCTCGGGTGGG (#2 in Fig. 2A)	*Cß* antisense	33–16^b^
5′‐TGCTCCTTGAGGGGCTGCG (#3 in Fig. 2A)	*Cß* antisense	196–178^b^
5′‐FAM‐TTCAGGTCCTCTCCAGGCACTG‐TAMRA (P in Fig. 2A)	*X1Cß* probe, antisense	straddling *X1* and *Cß*
5′‐ATTGCCGACAGGATGCAGAA	*ACTB* sense	998–1017^c^
5′‐GCTGGAAGGTGGACAGCGA	*ACTB* antisense	1133–1115^c^
5′‐TGCTCCTTGAGGGGCTGCG	*ACTB* probe	1053–1078^c^
5′‐GGAGGCTATCCAGCGTACT	*B2M* sense	114–132^c^
5′‐GACCAGTCCTTGCTGAAAGACA	*B2M* antisense	302–281^c^
5′‐CGGATGGATGAAACCCAGACACATAGC	*B2M* probe	220–194^c^
5′‐GCTTCACTGCTCAGGTGAT	*EEF1A1* sense	1079–1097^c^
5′‐GCCGTGTGGCAATCCAAT	*EEF1A1* antisense	1160–1143^c^
5′‐AAATAAGCGCCGGCTATGCCCCTG	*EEF1A1* probe	1118–1141^c^
5′‐GGTGTGAACCATGAGAAGTATGA	*GAPDH* sense	502–524^c^
5′‐GATGGCATGGACTGTGGTCA	*GAPDH* antisense	645–626^c^
5′‐CCTCAAGATCATCAGCAATGCCTCCTG	*GAPDH* probe	531–557^c^

To exclude genomic signals, in each amplicon either one primer or the probe straddles two exons. Double dye probes obtained from Eurogentec (Liège, Belgium) are 6‐FAM marked in 5′ and quenched in 3′ with TAMRA. All qPCR amplifications were performed on the same dT‐primed cDNA templates with sense primers located at similar distances from the poly‐A tail (785, 796, 874, 669, and 809 nt for *X1‐Cß*, *ACTB*, *B2M*, *EEF1A1*, and *GAPDH*, respectively).

^a^ Positions are relative to the TSS of exon *X1*.

^b^ Positions given from the 5′‐end of exon 1 of Cß1 and Cß2

^c^ Positions given according to the reference mRNAs: NM_001101.2 for *ACTB*; NM_004048 for *B2M*; NM_001402 for *EEF1A1*; NM_002046.3 for *GAPDH*.

### Detection of *X1‐Cß* transcripts by RT‐PCR

For the detection of *X1‐Cß* transcripts in T cell clones, Epstein‐Barr virus‐transformed B cell lines, tumor lines or fresh PBMC, RNA was extracted without DNAse pretreatment, by the TRIPure method (Roche®) and cDNA was readily obtained by reverse‐transcription of 1 μg of total RNA for 1 h at 42°C with 100 UI SmartScribe® RT of Clontech, the SMART II primer (1 μM) and an anchored‐dT_21_ primer (2.5 μM) in a volume of 10 μl. After an inactivation step at 70°C for 15 min, the cDNAs were diluted with water to 50 μl and stored at −20°C. PCR products of 227 bp were obtained from 2.5 μl of cDNA (derived from 50 ng RNA or 2500–10,000 cells) with primers 1 (*X1* sense), and 3 (*Cß* antisense, Fig. 2A and C and Table 1) and 0.625 UI of conventional Taq DNA polymerase (Takara) in a final volume of 25 μl, with 35 cycles (annealing at 60°C). These products were analyzed by gel electrophoresis and sequenced. To quantify the expression levels of *X1‐Cß*, amplified products of 64 bp were obtained from the same amounts of cDNA with primers 1 and 2 (Table 1 and Fig. 2A), Hot Start Taq DNA Polymerase (Eurogentec, Liège, Belgium), annealing and extension at 62°C on a StepOnePlus thermocycler (ABI), and quantified with a FAM‐TAMRA Double Dye probe (Eurogentec) straddling *X1* and *Cß* exon 1. Expression levels were normalized with *ACTB* and expressed as *X1‐Cß/ACTB* ratios obtained from ΔCq at identical thresholds, with verified amplification yields of 95% for both qPCR. We compared the levels of expression of the housekeeping genes *ACTB*, *GAPDH*, *EEF1A1,* and *B2M* in our samples 6 with the probe and primers indicated in Table 1, and observed the best correlations between the numbers of cells and *ACTB* expression levels.

### X1 promoter cloning

Gene segments of 1125, 943, 242, 95, and 48 bp located upstream of the major Transcription Start Site (TSS) of exon *X1* were obtained by PCR and cloned in Firefly luciferase vector pGL4.15 (Promega, Fitchburg, WI, USA), using Q5 (New England Biolabs) or the high fidelity DNA polymerase Pfu (Stratagene, La Jolla, CA, USA) mixed with Takara Taq. In the same experiments, we used a 187 bp sequence upstream of the *TRBV7.2* TSS, as a control promoter. In addition, a 400 bp fragment of the Enhancer ß (Eß) comprising the Eß‐core 7 was cloned in the unique BamH I site of pGL4.15 vectors containing the 943 bp fragment of the *X1* promoter or the 187 bp‐long *Vß7.2* promoter. The constructs were co‐transfected in triplicates in either HEK293T or Jurkat E6.1 cells with pGL4.75, a plasmid expressing the Renilla luciferase driven by a CMV promoter, as a transfection efficiency control. We used Lipofectamine 2000 (Invitrogen, Carlsbad, CA, USA) and *Trans*IT®Jurkat (Mirus Bio LLC) to transfect HEK293T and Jurkat cells, respectively. Luciferase activities were measured after 24–40 h using the Dual‐Glo® Luciferase Assay System (Promega) and a Glomax Discover plate reader (Promega). The Firefly luciferase activity was normalized to that of the Renilla luciferase and the results compared to those obtained with a promoterless vector, providing the ratios shown in Figure 3A.

### Isolation of human thymocytes

Postnatal thymuses were obtained from 0‐ to 12‐year‐old children that underwent cardiac surgery. Cord blood was obtained from the Navelstrengbloedbank UZ Gent. All human material was used following guidelines of the Medical Ethical Committee of the Ghent University Hospital (Belgium). Informed consent was obtained in accordance with the Declaration of Helsinki. A thymocyte suspension was made within 24 h after surgery. Cord blood mononuclear cells were obtained after density centrifugation. CD34^+^ cells were enriched by anti‐CD34 magnetic activated cell sorting (MACS Miltenyi Biotec) to a purity of >90% and subsequently labeled and sorted in CD34^+^CD1^−^CD3^−^CD45^+^ and CD34^+^CD1^+^CD3^−^CD45^+^. CD4iSP (CD1^+^CD45^+^CD4^+^CD3^−^CD8^−^) and DP (CD3^+^ CD4^+^CD8^+^) cells were sorted without pre‐enrichment procedures. Anti‐CD45, −CD34, −CD1, −CD3, −CD4, and −CD8a antibodies used for sorting were obtained from Miltenyi Biotech. The cells were sorted on a FACSAria II to a purity of >99%. RNA and cDNA were obtained as described above.

### Sequence analyses

To identify potential target sites of transcription factors within the X1 promoter, we used the JASPAR databank 2016, with a 0.85 threshold score (http://jaspar.genereg.net/cgi-bin/jaspar_db.pl?rm=browse&db=core&tax_group=vertebrates). DNase I Hypersensitive sites were predicted with the ENCODE project at http://www.genome.ucsc.edu/.

## Results

### A new germline *TCRB* transcript contains a previously undescribed exon

**Figure 1:**
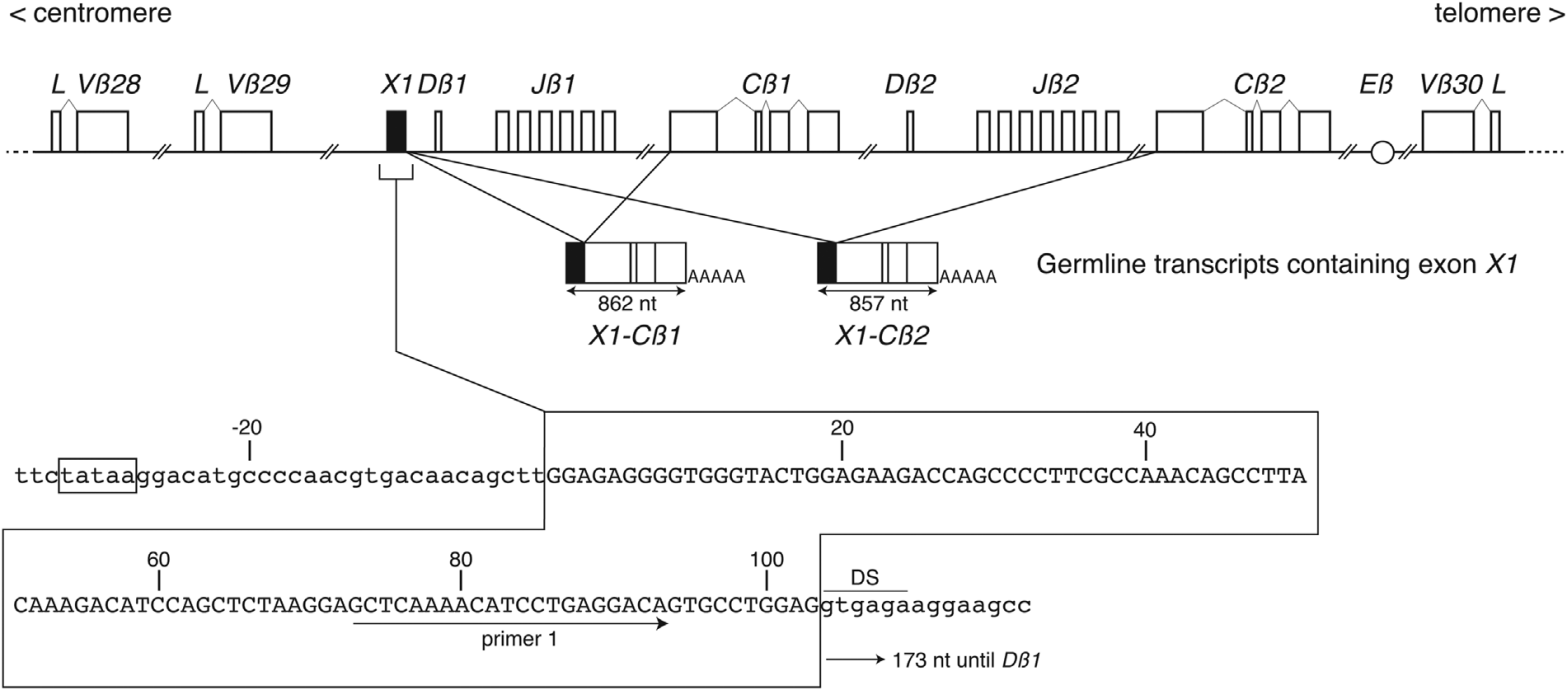
Exon *X1* within the human *TCRB* locus. Representation of the 3′ end of the unrearranged *TCRB* locus with the location of exon *X1*. The structure of the two most abundant germline *X1‐Cß* transcripts is indicated. The complete sequence of *X1* (103 bp) is boxed. A TATA box and a donor site of splicing (DS) are indicated. Primer 1 in *X1* was used in PCR amplifications. Sequences are accessible at European Nucleotide Archive: Exon *X1* (LT601549) and *X1‐Cß1* transcript (LT601550).

Starting from RNA extracted from eight melanomas that were infiltrated by T lymphocytes, we produced eight *TCRB* cDNA libraries using a TCR‐Cß reverse primer and the SMART oligonucleotide (Clontech). We sequenced about 1900 cDNA clones from these libraries. About 75% of the sequences corresponded to *TCRB* sequences: ±65% were in‐frame rearranged *TCRB* sequences with a median length of 420 bp, ±15% were *J‐C* sequences apparently initiated in front of *Jß2.3* (ENA LT626065) and other *Jß* gene segments, ±10% were very short *J‐C* products, and 5–10% were sequences of ±140 nucleotides, containing a *Cß1* (60%) or *Cß2* (40%) sequence preceded by 103 nucleotides not reported to be present in *TCRB* transcripts. In the human *TCRB* locus, this 103 bp sequence ends 174 nucleotides upstream of the *Dß1* gene segment. It is preceded by a potential TATA box and followed by a predicted 8 donor site of splicing (Fig. 1). These results suggested that this 103 nt sequence was the first exon, which we named *TRBX1* (*X1*), of a new germline *TCRB* transcript that contained *X1* and *Cß* sequences.

To identify the 3′ end of these *X1*‐containing transcripts, RNA was extracted from 3 T cell clones and reverse‐transcribed with an anchored oligo‐dT primer. The resulting cDNA was used as a template for a PCR amplification with the anchor and primer 1 in exon *X1* (Fig. 1) and the amplified products were sequenced. The sequences were about 860 bp long and corresponded to *X1* spliced either with the first exon of *Cß1* followed by *Cß1* exons 2–4, or with the first exon of *Cß2* followed by *Cß2* exons 2–4. All sequences had a 3′ polyA tail. The structure of the *X1‐Cß* transcripts is shown in Figure 1.

*X1‐Cß* gene products can only be transcribed from *TCRB* loci that have not undergone a *V‐DJ* rearrangement, as the latter deletes *X1* (Fig. 1). We surmised that *X1‐Cß1* transcripts originated from *TCRB* loci either in germline configuration or with *Dß1‐Jß1* rearrangements. Indeed, the first good acceptor site of splicing downstream of *X1* is that of *Cß1* exon 1. In case of *Dß1‐Jß2* rearrangements, *Cß1* is deleted and the next good acceptor site is that of *Cß2* exon 1. We confirmed this hypothesis by establishing the *TCRB* genomic structure and *Cß* usage of the *X1‐Cß* transcripts on a set of 12 T cell clones with a single *V‐DJ* rearrangement. As expected, no *X1‐Cß* transcripts were detected in the three clones with two *V‐DJ* rearrangements (data not shown).

Exon *X1* contains no ATG initiation codon and is therefore not expected to be translated. Translation of a protein from the *Cß* segment of *X1*‐*C*ß appears unlikely. The largest ORF is 141 nt long and ends 53 nt upstream of the exon 3/exon 4 junction. It is preceded by very short ORFs, a feature that disfavors its translation. In addition, translation ending in the second last exon at a distance greater than 50–55 nt from the last exon‐exon junction complex precludes the ribosome from removing the complex which then recruits RNAses and ubiquitin 9. We provisionally conclude that the *X1‐Cß* transcripts are sterile.

We conclude to the presence in human T lymphocytes of previously undescribed *TCRB* mRNAs that contain ±860 nt and consist of a new exon, *X1*, followed by *Cß1* or *Cß2* exons.

### Expression of *X1‐Cß* transcripts

**Figure 2:**
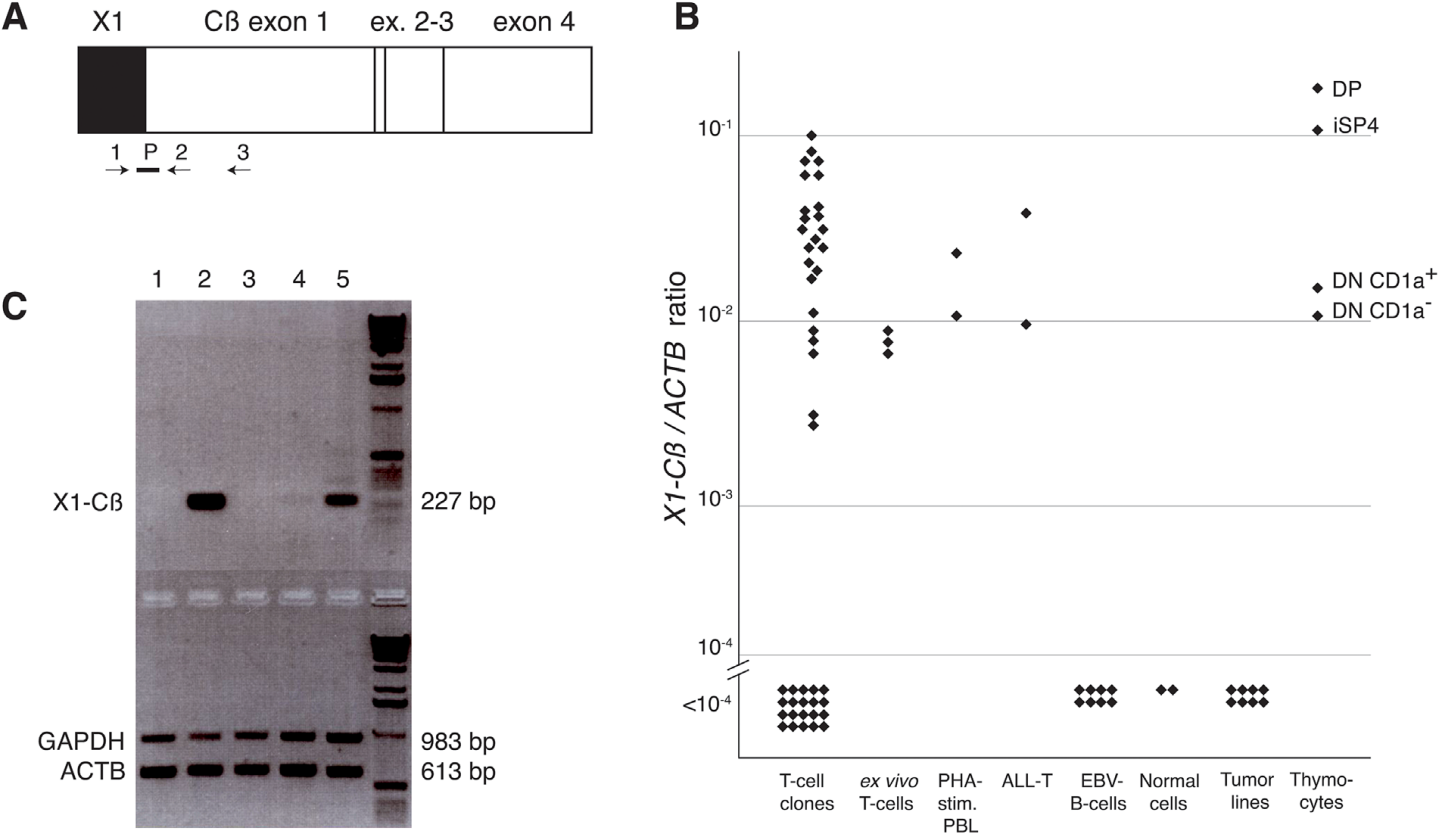
Expression of *X1‐Cß*. (A) Schematic representation of the primers used in quantitative (B) and conventional (C) *X1‐Cß* RT‐PCR assays. Arrows and bar indicate primers and probe listed in Table 1. (B) Levels of *X1‐Cß* expression were measured by RT‐qPCR using primers 1, 2, and probe P on cDNA obtained from T cell clones, freshly isolated T cells, Phytohemagglutinin‐A stimulated blood lymphocytes, acute T cell leukemias, Epstein‐Barr virus‐transformed B cells, fibroblasts, and CD34^+^ cord blood cells, tumor lines (7 melanomas and 1 sarcoma) and thymocytes at various maturation stages: CD4^+^CD8^+^ double positive (DP), immature CD4^+^ single positive (iSP4), CD4^−^CD8^−^ double negative (DN) CD1a^+^ or CD1a^−^. Results are presented as *X1‐Cß/ ACTB* ratios calculated as indicated in Materials and Methods. (C) Gel analysis of amplified products obtained with a conventional PCR using primers 1 and 3 on cDNA obtained from 5 T cell clones. RNA integrity was assessed by amplifying *GAPDH* and *ACTB* cDNAs in a parallel duplex PCR for 22 cycles. Lane 6 is a 1 kb ladder (Invitrogen).

We screened various cell types using a RT‐qPCR amplification with primers 1 and 2 located in *X1* and *Cß*, respectively, and a probe straddling the *X1‐Cß* junction (Fig. 2A and Table 1). From a set of 44 T cell clones previously established in our laboratory, only 24 (55%) expressed *X1‐Cß* transcripts (Fig. 2B). This proportion was expected: *X1* is lost during the *V‐DJ* recombination process and *X1‐Cß* transcripts are therefore absent from T cells that have undergone bi‐allelic *TCRB* rearrangements. In murine lymph node‐derived T cells, the proportion of cells with a single rearranged *TCRB* locus was estimated at 57% 10. *X1‐Cß* transcripts could also be detected in about 50% of T cell clones using a conventional RT‐PCR yielding a larger amplicon whose sequence could be verified (Fig. 2C). In cultured T cell clones, we estimated the levels of expression of *X1‐Cß* and complete *TCRB* transcripts at 2–10 and 100–300 mRNA molecules per cell, respectively 11 and data not shown). Thus in mature T cells the number of *X1‐Cß* transcripts is considerably lower than that of the *TCRB*‐encoding mRNAs.

We detected *X1‐Cß* expression in freshly isolated blood CD4 or CD8 T cells, in Phytohemagglutinin‐A activated blood T cells and in leukemic T cells including Jurkat E6.1. No expression was detected in non‐T cells such as fibroblasts, keratinocytes, monocytes, CD34^+^ cord blood cells, Epstein‐Barr virus‐transformed B cells and 35 tumor lines from various non‐T histological types (Fig. 2B and data not shown).

In sorted sub‐populations of thymocytes, *X1‐Cß* expression increased concurrent with ß‐selection: expression was low in pre‐ß selection CD34^+^CD1a^−^ and CD34^+^CD1a^+^ double negative (DN) cells (Fig. 2B) and 10‐times higher in post‐ß selection CD3^−^CD4^+^ (immature single positive cells: iSP4) and CD3^+^CD4^+^CD8^+^ DP populations (Fig. 2B).

We conclude that *X1‐Cß* transcripts are T cell‐specific and appear when *TCRB* gene rearrangements are initiated. They are absent from T cells with biallelic *V‐DJ* rearrangements.

### A promoter sequence in front of *X1*

**Figure 3:**
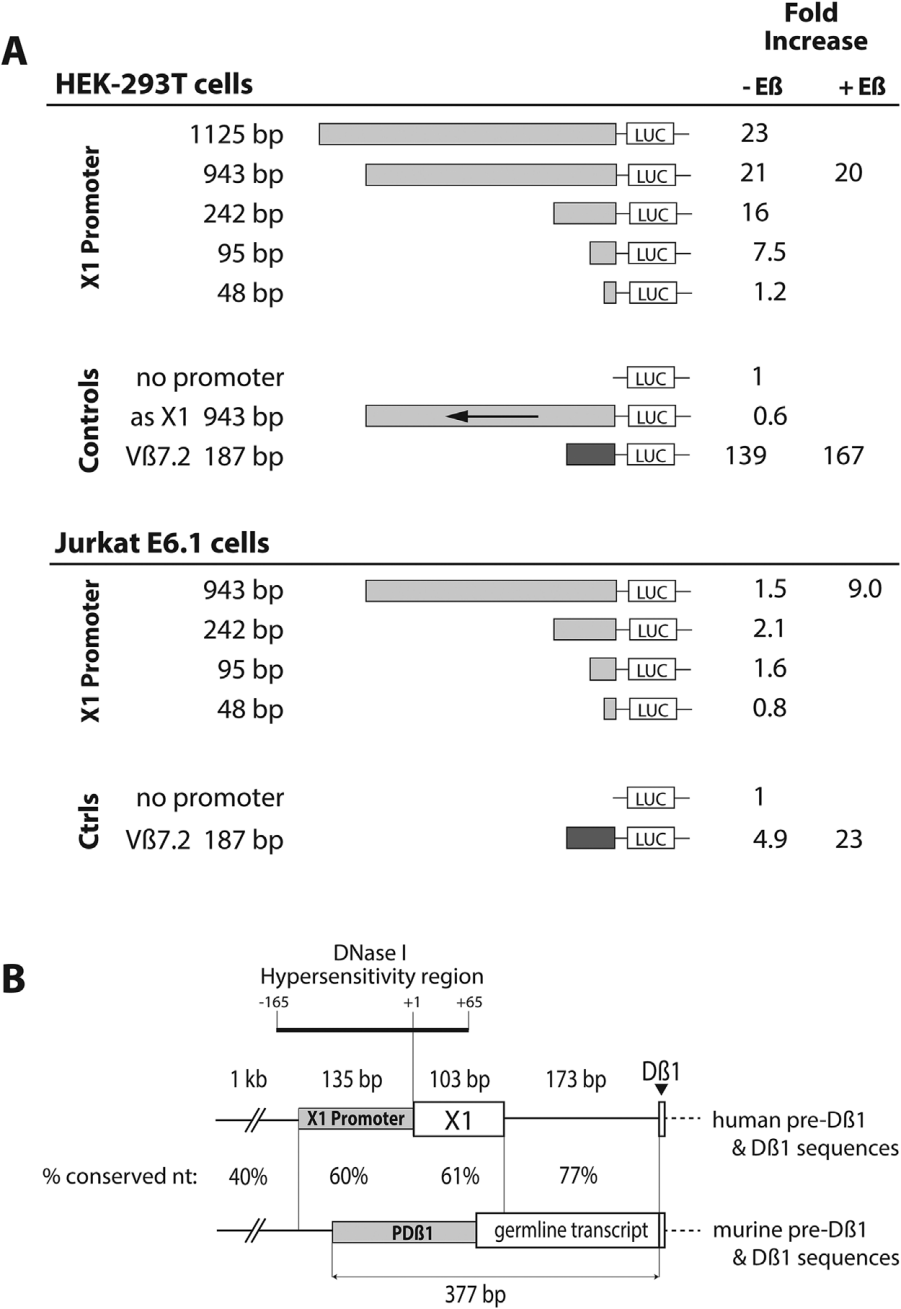
Characterization of the *X1* promoter region. (A) Genomic DNA fragments of indicated sizes, immediately preceding the TSS of exon *X1*, were cloned in front of the Firefly luciferase gene in vector pGL4.15, with or without *Eß* as indicated. The constructs were co‐transfected in HEK293T cells or Jurkat cells with vector pGL4.75 containing the Renilla luciferase sequence. Control constructs included pGL4.15 without promoter, with a *X1* promoter sequence cloned antisense (as) and with a *Vß7.2* promoter sequence. One day after transfection, both luciferase activities were measured. The results, means of 2–9 independent assays, are expressed relatively to those obtained with the pGL4.15 promoterless construct. Sequence of the 1125 bp promoter fragment: ENA LT601551. (B) Sequence homologies between the human and murine pre‐*Dß1* regions. Promoter and transcribed sequences are shown as closed and open boxes, respectively. The *PDB1* sequence proposed here is shorter at its 3′‐end than in the original description by Sikes 12, taking into account the longest germline transcripts reported by Doty 13 or present in Genbank (EST CB598216, and BB587363). The indicated DNase I hypersensitivity region straddling the TSS of *X1* is described for human T cells by the ENCODE project.

To examine the regulatory elements governing *X1* transcription, we cloned the genomic DNA immediately upstream of *X1* in a luciferase‐encoding vector and transfected the construct into HEK293T cells. We compared the promoter activities of five sequences of decreasing sizes (Fig. 3A). The highest promoter activities were observed for the two largest fragments, of 1125 and 943 bp, with similar inductions of 23‐ and 21‐fold versus that of the promoterless construct. These inductions were approximately sixfold lower than that observed with a *Vß7.2* promoter (Fig. 3A). An antisense construct of 943 bp had no activity (Fig. 3A). The shortest *X1* promoter fragment with a detectable activity was 95 bp long (7.5‐fold induction).

We also transfected several of these constructs in the leukemic T cell clone Jurkat E6.1, with or without the 393 bp core sequence of the *TCRB* gene enhancer (*Eß*) which was shown to enhance the transcription of several *TRBV* genes 7. Transfection of the 943 bp long *X1* promoter with *Eß* stimulated transcription (Fig. 3A). However, in the absence of *Eß* the stimulation of transcription was minimal, which was expected considering the role of *Eß* for *TCRB* transcription in T cells.

We conclude that a promoter sequence is present immediately upstream to the *X1* sequence. In line with this conclusion, a DNAse I hypersensitivity region straddling the TSS of *X1* is observed almost exclusively in T cells, according to the ENCODE project (Fig. 3B and Materials and Methods). Moreover, in T cells the X1 promoter is controlled by Eß.

The sequence of the *X1* promoter followed by *X1* appears to be the human ortholog of the murine *Dß1* Promoter (*PDß1)*, a 377 bp sequence located immediately upstream of the *TRBD1* gene segment 12 (Fig. 3B). Actually, this 377 bp sequence appears to consist in a promoter followed by the first 200 nt of germline transcripts controlled by this promoter. Indeed, reported transcripts contain the *PDß1* last 200 nt sequence followed by *Dß1* usually rearranged with a *Jß* spliced to a *Cß* segment 13 (and murine EST database). The homology between the human and murine DNA sequences upstream of *Dß1* is above 70% immediately 5′ to *Dß1*, remains at 60% for *X1,* and the last 135 bp of its promoter, then drops sharply to 40% for upstream sequences (Fig. 3B).

**Figure 4:**
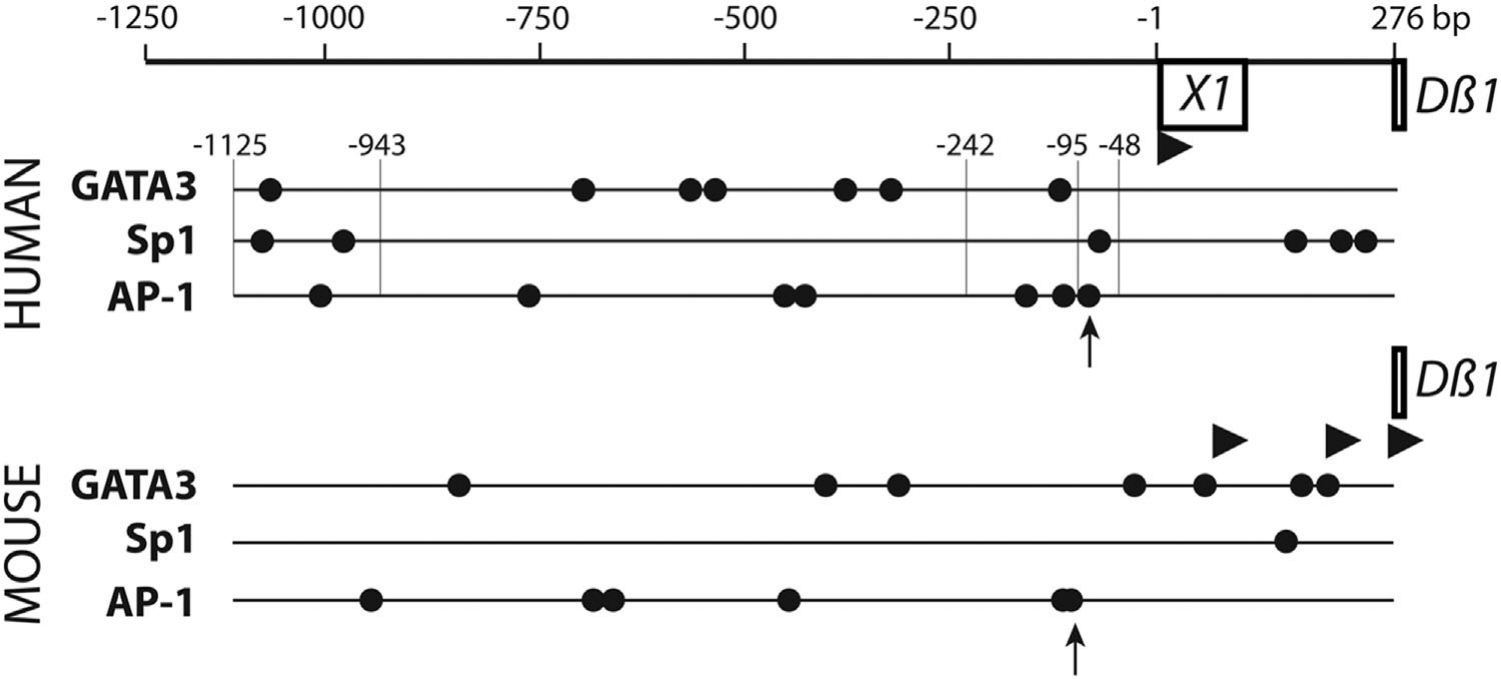
Transcription factor binding sites upstream of human and murine *Dß1*. Human and murine genomic DNA regions upstream of Dß1 are shown with predicted positions of transcription factors GATA3, SP1, and AP‐1 (scores ≥0.85 in the JASPAR data bank of target sequences for transcription factors of vertebrates, 2016). Arrow heads indicate reported starts of transcription. Vertical lines show the 5′‐ends of the *X1* promoter fragments analyzed in Figure 3A. Small arrows indicate an AP‐1 site present within a 16 nt stretch that is perfectly conserved between the human and murine sequences.

Sp1 and GATA3 have been shown to contribute to *PDß1* activity 12 and binding sites for these transcription factors are present also in the *X1* promoter. Seven GATA3 sites are present upstream of the human and murine *Dß1* sequences (Fig. 4). While a single and important Sp1 site is present in the mouse, three sites are present in the X1 promoter (Fig. 4). Interestingly, 16 nucleotides positioned 91–77 nt upstream to the TSS of X1 are perfectly conserved between human and murine sequences. They are contained in the 377 bp core PDß1 promoter reported by Sikes 12. They contain no GATA3 or Sp1 site, but a consensus AP‐1 site indicated with arrows on Figure 4. This AP‐1 site might participate together with an Sp1 site in the promoter activity present between nucleotides −48 and −95 of the human *X1* promoter (Fig. 3A).

## Discussion

In the mouse, several germline transcripts have been described that originate from the *IG* and *TCR* loci 14, 15, 16. They are usually expressed, when the gene segments from which they are derived are poised to rearrange 17. *IG* and *TCR* germline transcriptions have been considered as facilitating gene segment rearrangements by contributing to the accessibility of the loci to the recombinase complex 18, as deeply reviewed by Oltz and colleagues 19, 20.

For the *TCRB* locus, the best‐studied germline transcription occurs upstream of the murine *Dß1* gene segment and is controlled by a promoter named *PDß1* 12. The accessibility of *Jß1*, *Dß2‐Jß2,* and the proximal *Vß* gene segments is controlled by the enhancer *Eß* 14, but both *Eß* and *PDß1* are required for *Dß1‐Jß* rearrangements to occur 21. Deletion of *PDß1* or its displacement downstream toward *Jß1* was shown to prevent *Dß1‐Jß* recombinations 21, 22. However, *Dß1‐Jß* recombinations persisted after *PDß1* inversion, indicating that it is not the germline transcription through the *Jß* segments that is important for *Dß1‐Jß* recombination 22. Finally, mutations of GATA3 or Sp1 binding sites within *PDß1* strongly impaired *Dß1* recombinations 12, 13. Thus, like *Eß*, *PDß1* is an *accessibility control element* (ACE), even though it controls a much shorter genomic interval than *Eß* does. The accepted model is that the interaction of *PDß1* with *Eß* leads to the recruitment of additional factors that ultimately favor recombination by locally reorganizing the chromatin structure. One of these factors is a component of the SWI/SNF complex, which is required in murine thymocytes to open the *Dß1* region prior to recombination 23.

Very little is known about *IG* and *TCR* germline transcription in human cells 24, 25. We describe a new germline transcript from the human *TCRB* locus, with a new exon, *X1*, located upstream of *Dß1* and spliced with the first exon of *Cß1* or *Cß2*. *X1‐Cß* transcription is controlled by a promoter that appears to be the ortholog of *PDß1*, for two reasons. First, both are localized in front of germline transcripts initiated in the pre‐Dß1 region. Indeed in the mouse, the *PDß1* sequences described by Sikes 12 and Whitehurst 21 contain more than a promoter, as their 3′ halves can be transcribed 13 (and Genbank EST database). Accordingly the core promoter in PDß1 is less than 200 bp long and starts 377 bp upstream of *Dß1* (Fig. 3B). In the human *TCRB* locus, the shortest tested sequence with *X1*‐promoter activity is 95 bp long and starts 371 bp upstream of *Dß1*. Second, the 410 nucleotides upstream of murine and human *Dß1* are homologous. The homology is maximal (77%) next to *Dß1*, is maintained at about 60% until the 5′ end of the core promoters, then drops sharply to 40% (Fig. 3B).

In line with their role of ACEs, the murine *PDß1* and the human *X1* promoter are activated at the earliest stages of thymocyte maturation, that is prior to *Dß* rearrangements. Indeed, *PDß1*‐controlled transcripts have been detected in thymocytes of Rag^−/−^ animals 13, 14, and we have detected *X1‐Cß* transcripts in human CD34^+^ CD1a^−^ CD4^−^ CD8^−^ CD45^+^ (DN) thymocytes, which contain no proteins with a Cß1 domain 26 and very few if any *Dß‐Jß* recombination products 2.

Our results suggest that *X1‐Cß* expression is 10‐times higher in late stages of thymocyte differentiation (iSP4 and DP) than in earlier stages (DN). The timeframe for *V‐DJ* rearrangements at the human *TCRB* locus extends until the DP stage 2, 3, 26, 27. Accordingly, a high level of *X1‐Cß* transcription at the time of *V‐DJ* recombination may contribute to this stochastic process by increasing the accessibility of the 5′RSS of the recombining *Dß1* gene segment. Mutations in *X1* promoter might cause biases in the TCR repertoire, with a higher proportion of *Vß‐Dß2* rearrangements.

We observed that *X1‐Cß* expression persisted in mature T cells without two complete *TCRB* rearrangements. This persistence contrasts with what has been observed for other *IG* or *TCR* germline transcripts such as *J‐Ck*, *I‐Cμ*, T early alpha (*TEA*), and *Vß*, which are expressed mainly in immature lymphocytes 18, 28, 29.

*X1‐Cß* was also expressed in some acute B cell leukemias (data not shown). This expression is linked to an accessible *TCRB* locus leading to an incomplete ß‐rearrangement process 30, 31, 32. *X1‐Cß* is expressed at low levels in the erythroleukemia line K562, which is one of the rare non‐T cell lines with some DNase I hypersensitivity around the TSS of *X1* (ENCODE project).

Allelic inclusion at the *TCRB* locus, that is leading to the presence of two functional TCRß chains on the cell surface, has been reported to occur in about 1% of human T cell clones, suggesting that allelic exclusion at this locus is not an absolute rule 33, 34. The difficulty of these genetic analyses is to ensure that the analyzed cell populations are clonal. In this context, an RT‐PCR assay for *X1‐Cß* can help: in a given clone the detection of *X1‐Cß* transcripts excludes the presence of two complete TCRß rearrangements (except for the rare *Vß30‐Dß2‐Jß2* rearrangements).

## Conflict of Interest

The authors declare no commercial or financial conflict of interest.

## References

[1] Spicuglia, S. , A. Pekowska , J. Zacarias‐Cabeza , and P. Ferrier . 2010 Epigenetic control of Tcrb gene rearrangement. Semin. Immunol. 22(6):330–336. 20829066 10.1016/j.smim.2010.07.002

[2] Dik, W. A. , K. Pike‐Overzet , F. Weerkamp , D. de Ridder , E. F. de Haas , M. R. Baert , P. van der Spek , E. E. Koster , M. J. Reinders , J. J. van Dongen , et al. 2005 New insights on human T cell development by quantitative T cell receptor gene rearrangement studies and gene expression profiling. J. Exp. Med. 201(11):1715–1723. 15928199 10.1084/jem.20042524 PMC2213269

[3] Taghon, T. , I. Van de Walle , G. De Smet , M. De Smedt , G. Leclercq , B. Vandekerckhove , and J. Plum . 2009 Notch signaling is required for proliferation but not for differentiation at a well‐defined beta‐selection checkpoint during human T‐cell development. Blood 113(14):3254–3263. 18948571 10.1182/blood-2008-07-168906

[4] Lurquin, C. , B. Lethé , E. De Plaen , V. Corbière , I. Théate , N. van Baren , P. G. Coulie , and T. Boon . 2005 Contrasting frequencies of antitumor and anti‐vaccine T cells in metastases of a melanoma patient vaccinated with a MAGE tumor antigen. J. Exp. Med. 201(2):249–257. 15657294 10.1084/jem.20041378 PMC2212799

[5] Matz, M. , D. Shagin , E. Bogdanova , O. Britanova , S. Lukyanov , L. Diatchenko , and A. Chenchik . 1999 Amplification of cDNA ends based on template‐switching effect and step‐out PCR. Nucleic Acids Res. 27(6):1558–1560. 10037822 10.1093/nar/27.6.1558 PMC148354

[6] Bustin, S. A. , V. Benes , J. A. Garson , J. Hellemans , J. Huggett , M. Kubista , R. Mueller , T. Nolan , M. W. Pfaffl , G. L. Shipley , et al. 2009 The MIQE guidelines: minimum information for publication of quantitative real‐time PCR experiments. Clin. Chem. 55(4):611–622. 19246619 10.1373/clinchem.2008.112797

[7] Gottschalk, L. R. , and J. M. Leiden . 1990 Identification and functional characterization of the human T‐cell receptor beta gene transcriptional enhancer: common nuclear proteins interact with the transcriptional regulatory elements of the T‐cell receptor alpha and beta genes. Mol. Cell. Biol. 10(10):5486–5495. 2144610 10.1128/mcb.10.10.5486-5495.1990 PMC361259

[8] Senapathy, P. , M. B. Shapiro , and N. L. Harris . 1990 Splice junctions, branch point sites, and exons: sequence statistics, identification, and applications to genome project. Methods Enzymol. 183:252–278. 2314278 10.1016/0076-6879(90)83018-5

[9] Schweingruber, C. , S. C. Rufener , D. Zund , A. Yamashita , and O. Muhlemann . 2013 Nonsense‐mediated mRNA decay—mechanisms of substrate mRNA recognition and degradation in mammalian cells. Biochim. Biophys. Acta 1829(6–7):612–623. 23435113 10.1016/j.bbagrm.2013.02.005

[10] Khor, B. , and B. P. Sleckman . 2005 Intra‐ and inter‐allelic ordering of T cell receptor beta chain gene assembly. Eur. J. Immunol. 35(3):964–970. 15719363 10.1002/eji.200425806

[11] Lennon, G. P. , J. E. Sillibourne , E. Furrie , M. J. Doherty , and R. A. Kay . 2000 Antigen triggering selectively increases TCRBV gene transcription. J. Immunol. 165(4):2020–2027. 10925285 10.4049/jimmunol.165.4.2020

[12] Sikes, M. L. , R. J. Gomez , J. Song , and E. M. Oltz . 1998 A developmental stage‐specific promoter directs germline transcription of D beta J beta gene segments in precursor T lymphocytes. J. Immunol. 161(3):1399–1405. 9686603

[13] Doty, R. T. , D. Xia , S. P. Nguyen , T. R. Hathaway , and D. M. Willerford . 1999 Promoter element for transcription of unrearranged T‐cell receptor beta‐chain gene in pro‐T cells. Blood 93(9):3017–3025. 10216098

[14] Mathieu, N. , W. M. Hempel , S. Spicuglia , C. Verthuy , and P. Ferrier . 2000 Chromatin remodeling by the T cell receptor (TCR)‐beta gene enhancer during early T cell development: implications for the control of TCR‐beta locus recombination. J. Exp. Med. 192(5):625–636. 10974029 10.1084/jem.192.5.625 PMC2193263

[15] Engel, H. , H. Ruhl , C. J. Benham , J. Bode , and S. Weiss . 2001 Germ‐line transcripts of the immunoglobulin lambda J‐C clusters in the mouse: characterization of the initiation sites and regulatory elements. Mol. Immunol. 38(4):289–302. 11566322 10.1016/s0161-5890(01)00056-6

[16] Abarrategui, I. , and M. S. Krangel . 2009 Germline transcription: a key regulator of accessibility and recombination. Adv. Exp. Med. Biol. 650:93–102. 19731804 10.1007/978-1-4419-0296-2_8

[17] Schlissel, M. S. , and P. Stanhope‐Baker . 1997 Accessibility and the developmental regulation of V(D)J recombination. Semin. Immunol. 9(3):161–170. 9200327 10.1006/smim.1997.0066

[18] Yancopoulos, G. D. , and F. W. Alt . 1985 Developmentally controlled and tissue‐specific expression of unrearranged VH gene segments. Cell 40(2):271–281. 2578321 10.1016/0092-8674(85)90141-2

[19] Cobb, R. M. , K. J. Oestreich , O. A. Osipovich , and E. M. Oltz . 2006 Accessibility control of V(D)J recombination. Adv. Immunol. 91:45–109. 16938538 10.1016/S0065-2776(06)91002-5

[20] Thomas, L. R. , R. M. Cobb , and E. M. Oltz . 2009 Dynamic regulation of antigen receptor gene assembly. Adv. Exp. Med. Biol. 650:103–115. 19731805 10.1007/978-1-4419-0296-2_9

[21] Whitehurst, C. E. , M. S. Schlissel , and J. Chen . 2000 Deletion of germline promoter PD beta 1 from the TCR beta locus causes hypermethylation that impairs D beta 1 recombination by multiple mechanisms. Immunity 13(5):703–714. 11114382 10.1016/s1074-7613(00)00069-8

[22] Sikes, M. L. , A. Meade , R. Tripathi , M. S. Krangel , and E. M. Oltz . 2002 Regulation of V(D)J recombination: a dominant role for promoter positioning in gene segment accessibility. Proc. Natl. Acad. Sci. U. S. A. 99(19):12309–12314. 12196630 10.1073/pnas.182166699 PMC129441

[23] Osipovich, O. , R. M. Cobb , K. J. Oestreich , S. Pierce , P. Ferrier , and E. M. Oltz . 2007 Essential function for SWI‐SNF chromatin‐remodeling complexes in the promoter‐directed assembly of Tcrb genes. Nat. Immunol. 8(8):809–816. 17589511 10.1038/ni1481

[24] Calman, A. F. , and B. M. Peterlin . 1986 Expression of T cell receptor genes in human B cells. J. Exp. Med. 164(6):1940–1957. 2431093 10.1084/jem.164.6.1940 PMC2188500

[25] Berman, J. E. , C. G. Humphries , J. Barth , F. W. Alt , and P. W. Tucker . 1991 Structure and expression of human germline VH transcripts. J. Exp. Med. 173(6):1529–1535. 1903431 10.1084/jem.173.6.1529 PMC2190842

[26] Joachims, M. L. , J. L. Chain , S. W. Hooker , C. J. Knott‐Craig , and L. F. Thompson . 2006 Human alpha beta and gamma delta thymocyte development: tCR gene rearrangements, intracellular TCR beta expression, and gamma delta developmental potential‐differences between men and mice. J. Immunol. 176(3):1543–1552. 16424183 10.4049/jimmunol.176.3.1543 PMC1592528

[27] Ramiro, A. R. , C. Trigueros , C. Marquez , J. L. San Millan , and M. L. Toribio . 1996 Regulation of pre‐T cell receptor (pT alpha‐TCR beta) gene expression during human thymic development. J. Exp. Med. 184(2):519–530. 8760805 10.1084/jem.184.2.519 PMC2192728

[28] Duber, S. , H. Engel , A. Rolink , K. Kretschmer , and S. Weiss . 2003 Germline transcripts of immunoglobulin light chain variable regions are structurally diverse and differentially expressed. Mol. Immunol. 40(8):509–516. 14563370 10.1016/s0161-5890(03)00226-8

[29] de Villartay, J. P. , D. Lewis , R. Hockett , T. A. Waldmann , S. J. Korsmeyer , and D. I. Cohen . 1987 Deletional rearrangement in the human T‐cell receptor alpha‐chain locus. Proc. Natl. Acad. Sci. U. S. A. 84(23):8608–8612. 3500476 10.1073/pnas.84.23.8608 PMC299594

[30] Pelicci, P. G. , D. M. Knowles, 2nd , and R. Dalla Favera . 1985 Lymphoid tumors displaying rearrangements of both immunoglobulin and T cell receptor genes. J. Exp. Med. 162(3):1015–1024. 3875679 10.1084/jem.162.3.1015 PMC2187802

[31] Dombret, H. , P. Loiseau , J. C. Bories , and F. Sigaux . 1992 Unexpected consistent involvement of V beta gene segments in inappropriate T‐cell receptor beta gene rearrangements occurring in B‐lineage acute lymphoblastic leukemias. Blood 80(10):2614–2621. 1330077

[32] Szczepanski, T. , M. J. Pongers‐Willemse , A. W. Langerak , and J. J. van Dongen . 1999 Unusual immunoglobulin and T‐cell receptor gene rearrangement patterns in acute lymphoblastic leukemias. Curr. Top. Microbiol. Immunol. 246:205–213. Discussion 14–5. 10396058 10.1007/978-3-642-60162-0_26

[33] Davodeau, F. , M. A. Peyrat , F. Romagne , A. Necker , M. M. Hallet , H. Vie , and M. Bonneville . 1995 Dual T cell receptor beta chain expression on human T lymphocytes. J. Exp. Med. 181(4):1391–1398. 7699325 10.1084/jem.181.4.1391 PMC2191978

[34] Padovan, E. , C. Giachino , M. Cella , S. Valitutti , O. Acuto , and A. Lanzavecchia . 1995 Normal T lymphocytes can express two different T cell receptor beta chains: implications for the mechanism of allelic exclusion. J. Exp. Med. 181(4):1587–1591. 7699339 10.1084/jem.181.4.1587 PMC2191970

